# Chip Implementation with a Combined Wireless Temperature Sensor and Reference Devices Based on the DZTC Principle

**DOI:** 10.3390/s111110308

**Published:** 2011-10-31

**Authors:** Ming-Hui Chang, Yu-Jie Huang, Han-Pang Huang, Shey-Shi Lu

**Affiliations:** 1 Department of Mechanical Engineering, National Taiwan University, Taipei, 106, Taiwan; E-Mail: d93522037@ntu.edu.tw; 2 Department of Electrical Engineering, National Taiwan University, Taipei, 106, Taiwan; E-Mails: f95943067@ntu.edu.tw (Y.-J.H.); sslu@ntu.edu.tw (S.-S.L.)

**Keywords:** DZTC, temperature sensor, SAR ADC

## Abstract

This paper presents a novel CMOS wireless temperature sensor design in order to improve the sensitivity and linearity of our previous work on such devices. Based on the principle of CMOS double zero temperature coefficient (DZTC) points, a combined device is first created at the chip level with two voltage references, one current reference, and one temperature sensor. It was successfully fabricated using the 0.35 μm CMOS process. According to the chip results in a wide temperature range from −20 °C to 120 °C, two voltage references can provide temperature-stable outputs of 823 mV and 1,265 mV with maximum deviations of 0.2 mV and 8.9 mV, respectively. The result for the current reference gives a measurement of 23.5 μA, with a maximum deviation of 1.2 μA. The measurements also show that the wireless temperature sensor has good sensitivity of 9.55 mV/°C and high linearity of 97%. The proposed temperature sensor has 4.15-times better sensitivity than the previous design. Moreover, to facilitate temperature data collection, standard wireless data transmission is chosen; therefore, an 8-bit successive-approximation-register (SAR) analog-to-digital converter (ADC) and a 433 MHz wireless transmitter are also integrated in this chip. Sensing data from different places can be collected remotely avoiding the need for complex wire lines.

## 1. Introduction

Reference devices, such as voltage and/or current references, are key elements in many mixed-signal and analog applications. They are required to be stable throughout the process and to not be susceptible to power supply voltage, and temperature variations. In particular, in the system-on-a-chip (SoC) era, millions of transistors greatly increase the power dissipation. A chip with a built-in temperature sensor increases the system’s reliability by predicting fatal faults caused by excessive chip temperature. That means if the chip temperature is over the limit, the sensor can signal the chip power management unit to enter into the power-saving mode or give a warning.

With the rapid evolution of CMOS technology, CMOS bandgap references were developed [1,2], but only parasitic bipolar junction transistors (BJTs) can be used. Lakdawala [3] used the ratio of currents driven into a BJT pair with current chopping to up-convert the temperature signal and cancel the effects of parasitic resistance. For full CMOS voltage references, Najafizadeh [4] used the proportional to the absolute temperature (PTAT) current source to bias a diode-connected transistor to achieve a temperature-independent voltage reference, but there were no chip results to justify the claims. The M3 transistor suffers from a body effect because its source terminal is connected to a resistor. Zito [5] also used the PTAT sensing element to detect different emitter currents, but the device can only measure temperatures ranging from 20 °C to 100 °C. Smart temperature sensors [6–8] based on parasitic bipolar transistors display inaccuracies as low as a few tenths of a degree over the military temperature range, *i.e.*, from −55 °C to 125 °C, but require a one-point trim. Another way [9] was to bias a diode-connected transistor at a zero temperature coefficient (ZTC) point by using a constant current, but the realization of a temperature-independent current source was difficult. Although the combined design of the voltage reference and the temperature sensor was proposed in [10], only simulation results were available. In our previous design [11], based on the CMOS PTAT principle, a combined device for voltage reference and temperature sensors was successfully implemented using a fully digital process. For the temperature range from 20 °C to 120 °C, the experimental results showed that the voltage reference has a temperature stable output of 717 mV and the associated temperature sensor has the sensitivity of 2.3 mV/°C with linearity up to 95%. In order to improve the measurement range, linearity, and sensitivity of our previous design using the PTAT principle, a new DZTC-based temperature sensor design is proposed for performance enhancement. According to the chip results, the new design [12] can achieves better sensitivity and linearity than the one described in our previous work. For standalone applications, the device gives an analog output and provides digital output with embedded successive-approximation-register (SAR) analog-to-digital converter (ADC).

Here, a novel CMOS wireless temperature sensor is designed in order to improve the sensitivity and linearity of our previous design. Based on the principle of CMOS DZTC points, a combined device is first created at the chip level with two voltage references, one current reference, and one temperature sensor. In addition, with the integrated wireless transmitter, sensing temperature data from the chip can be transmitted to a data collector through a standard wireless approach.

This paper is organized as follows: Section 2 describes the system architecture. Section 3 introduces the circuit design of the proposed wireless temperature sensor. The experimental results are presented in Section 4. Finally, conclusions are given in Section 5.

## 2. System Architecture

**Figure 1.**
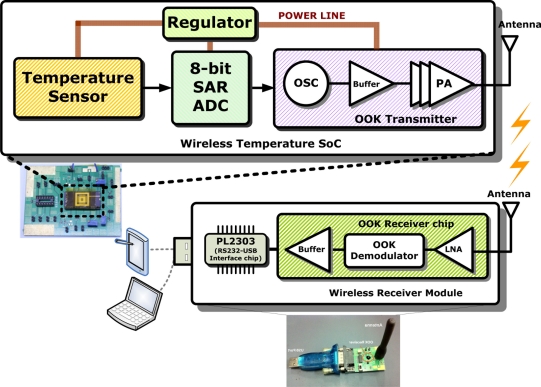
Block diagram of the proposed architecture.

A block diagram of the proposed architecture is given in Figure 1. The architecture is mainly divided into four parts: the temperature sensor, 8-bit SAR ADC, on-off keying (OOK) transmitter, and the regulator. The temperature sensor block consists of two voltage references, one current reference, and one temperature sensor. During operation, the surrounding temperature is converted into a voltage signal by the temperature sensor. Subsequently, the magnitude of the voltage signal is converted into serial digital data in an RS-232 format by an 8-bit SAR ADC, and then is wirelessly transmitted by the 433 MHz OOK transmitter. Finally, the data collectors, such as laptops and personal digital assistants (PDAs), can acquire and store immediate data through the developed wireless receiver module. In addition, data analysis can also be carried out by the software.

## 3. Circuit Design

### 3.1. Temperature Sensor

#### 

##### A. ZTC Point

Considering a diode-connected NMOS transistor exactly biased at the ZTC point with a constant drain current *I_D,ZTC_* for *T* = *T*_0_, its gate-source voltage can be written as:

(1) $$V_{\mathrm{GS}}\left(T_{0}\right)\equiv V_{\mathrm{GS,ZTC}}=V_{\mathrm{TH}0}+V_{\mathrm{OD}0}=V_{\mathrm{TH}0}+\sqrt{\frac{2I_{\mathrm{D,ZTC}}}{\mu_{0}C_{\mathrm{ox}}\left(W/L\right)}}$$

where *V_GS,ZTC_*, *I_D,ZTC_*, and *V*_*OD*0_ are the gate-source voltage, drain current, and overdrive voltage for such a transistor biased at the ZTC point, respectively. *W* and *L* are the channel width and length of the device, respectively. *C_ox_* is the oxide capacitance per unit area from gate to channel. *V*_*TH*0_ and *μ*_0_ are the threshold voltage and mobility at *T* = *T*_0_, respectively. For an arbitrary temperature, *T*, the gate-source voltage of this diode-connected transistor biased at the same drain current of *I_D,ZTC_* is represented as [13]:

(2) $$V_{\mathrm{GS}}\left(T\right)=V_{\mathrm{TH}}\left(T\right)+\sqrt{\frac{2I_{\mathrm{D,ZTC}}}{\mu \left(T\right)C_{\mathrm{ox}}\left(W/L\right)}}=V_{\mathrm{TH}0}+K_{T1}\left(\frac{T-T_{0}}{T_{0}}\right)+\sqrt{\frac{2I_{\mathrm{D,ZTC}}}{\mu_{0}C_{0x}\left(W/L\right)}}{\left(1+\frac{T-T_{0}}{T_{0}}\right)}^{\frac{-U_{\mathrm{TE}}}{2}}$$

Let the gate-source voltage of Equation (2) be independent of temperature, and differentiate *V_GS_*(*T*) with respect to *T* and assume *U_TE_* is exactly equal to −2. Then it is easy to prove the following identity for all *T*:

(3) $$V_{\mathrm{GS}}\left(T\right)=V_{\mathrm{TH}}\left(T\right)+V_{\mathrm{od}}\left(T\right)=V_{\mathrm{TH}0}+V_{\mathrm{OD}0}=V_{\mathrm{GS}}\left(T_{0}\right)\equiv V_{\mathrm{GS,ZTC}}$$

The typical value of parameter *U_TE_*, is −1.5. However, in modern technologies, *U_TE_* = −2 is achievable for n-channel devices by adjusting the dopant concentrations of *N_A_* and *N_D_* [4]. The I-V curve for a diode-connected NMOS with *U_TE_* = −2 exists at a unique ZTC point, but for *U_TE_* = −1.5, there is no common intersection point. It exists in a bottleneck only.

##### B. Double ZTC Voltage and Current Reference

**Figure 2.**
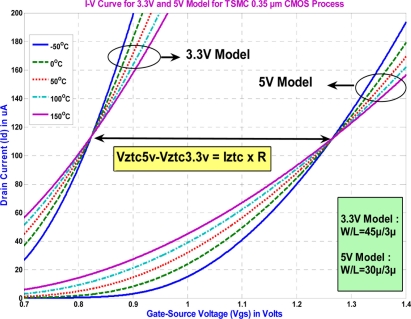
The design concept for DZTC voltage and current references.

TSMC 0.35 μm CMOS technology not only provides the 3.3 V transistor model with thin gate-oxide, but also gives the 5 V transistor model with thick gate-oxide. If both can have their *U_TE_* at about −2, there exist two unique ZTC points simultaneously. One is for the 3.3 V model and the other is for the 5 V model, as shown in Figure 2.

Fortunately, the NMOS models, for the channel length and width in the range of 12 *μ*m ≤ (*L,W*) ≤ 20 *μ*m, read *U_TE_* = − 2.06 for the 3.3 V model, and *U_TE_* = − 1.82 for the 5 V model. Both ZTC points are shown in Figure 2. Using these two ZTC points, the DZTC voltage and current references can be designed for temperature independency. Assuming that the resistor, *R*, is less sensitive with respect to temperature, the IR drop equals the difference of both ZTC voltages:

(4) $$\Delta V=V_{\mathrm{ZTC},5V}-V_{\mathrm{ZTC},3.3V}=I_{\mathrm{D,ZTC}}R$$

**Figure 3.**
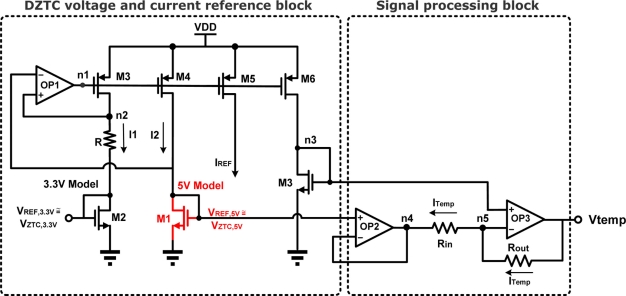
DZTC voltage, current reference, and temperature sensor.

The left portion of the block shown in Figure 3 is utilized to realize the concept of DZTC voltage and current references. In this block, all transistors use the 3.3 V CMOS model, except M1 which uses the 5 V model. To improve the circuit stability, the operational amplifier OP1 in Figure 3 must provide a sufficient stability margin. It uses folded-cascode topology and provides about a 52 dB gain, a 2 MHz gain-bandwidth, and an 87° phase margin. Since both nodes of n2 and *V*_*REF*,5*V*_ are connected to the inputs of OP1, they have the same voltage. The output of OP1 adjusts the current to suitably bias the transistors of M1 and M2 at their ZTC points according to the relationship of Equation (4). The output voltage for M1 is denoted as *V*_*REF*,5*V*_ with a value of about *V*_*ZTC*,5*V*_, and for M2 as *V*_*REF*,3.3*V*_ with a value of about *V*_*ZTC*,3.3*V*_. For the resistor, *R*, with less temperature sensitivity, the topology gives two reference voltages and a temperature-invariant current source. All of these are temperature-invariant.

##### C. Sensitivity Enhancement of Temperature Sensor

The right portion of the block shown in Figure 3 is used to realize the temperature sensor. The temperature information is obtained from nodes n3 and *V*_*REF*,5*V*_ in Figure 3. A unity-gain buffer, OP2, is utilized for isolation purposes. The other OP3 is used to amplify the signal and to improve the linearity with respect to temperature. Assume the open-loop gains of OP2 and OP3 are infinite. The closed-loop gain arising from OP3 becomes:

(5) $$\left|A_{\mathrm{cl}}\left(T\right)\right|=\frac{R_{\mathrm{out}0}\left[1+\gamma \left(T-T_{0}\right)\right]}{R_{\mathrm{in}0}\left[1+\gamma \left(T-T_{0}\right)\right]}=\left|A_{\mathrm{cl}}\right|$$

where γ is temperature coefficient of the resistor *R*, *R*(*T*) ≅ *R*_0_[1 + γ(*T* − *T*_0_)]. Clearly, if both resistors *R_in_* and *R_out_* use the same materials, the gain is independent of temperature. The temperature probed at node *V_Temp_* yields:

(6) $$V_{\mathrm{Temp}}\left(T\right)=V_{\mathrm{GS}3}\left(T\right)+I_{\mathrm{Temp}}R_{\mathrm{out}}\left(T\right)=V_{\mathrm{GS}3}\left(T\right)+\left(V_{\mathrm{GS}3}\left(T\right)-V_{\mathrm{ZTC},5V}\right)\times \left|A_{\mathrm{cl}}\right|$$

The sensitivity of the temperature sensor is calculated by:

(7) $$S=\frac{V_{\mathrm{Temp},\text{max}}-V_{\mathrm{Temp},\text{min}}}{T_{\text{max}}-T_{\text{min}}}$$

where *T_max_* is maximum temperature (120 °C) of measurement, *T_min_* is the minimum temperature measurement (−20 °C), *V_Temp,max_* is the output voltage of temperature sensor when the temperature is 120 °C, *V_Temp,min_* is the output voltage of temperature sensor when the temperature is −20 °C.

##### D. Temperature Variation of the Resistor

**Figure 4.**
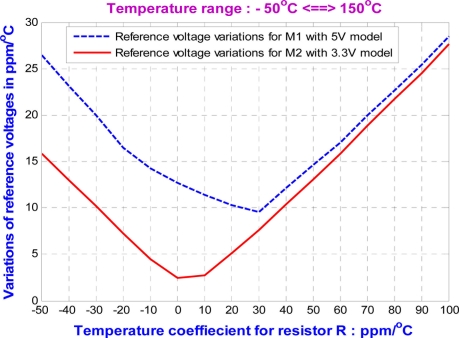
The variations of voltage reference due to the temperature variation of *R*.

**Figure 5.**
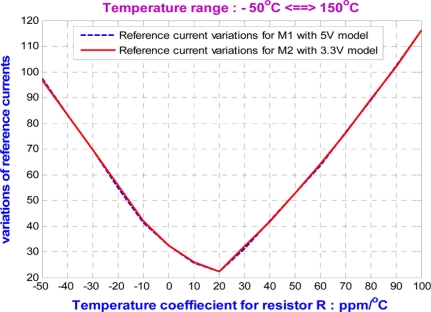
The variations of current source due to the temperature variation of *R*.

The temperature variation of the resistor, *R*, has an influence on the stability of the DZTC voltage and current references for wide temperature operations. Sweeping the temperature variations of the resistor, *R*, from −50 ppm/°C to 100 ppm/°C, both output reference voltages show temperature variations of less than 30 ppm/°C, as shown in Figure 4. Furthermore, the reference current source keeps the temperature variation below 120 ppm/°C, as shown in Figure 5. Obviously, the voltage reference is more stable than the current reference for large temperature variations of the resistor, *R*. In fact, if the temperature variation of the resistor, *R*, is small, the DZTC voltage and current references provide a reference current source and two reference voltages with very low temperature dependency.

The accuracy of the proposed DZTC voltage and current references depend on the temperature variation of the resistor, *R*. Fortunately, some commercial resistor products with low temperature coefficient can be obtained, such as the metal film resistor possibly achieving temperature coefficients of 10 ppm/°C. For applications, this design can use off-chip connection of the metal film resistor to achieve highly temperature-independent voltage and current references.

##### E. Monte Carlo Analysis

**Figure 6.**
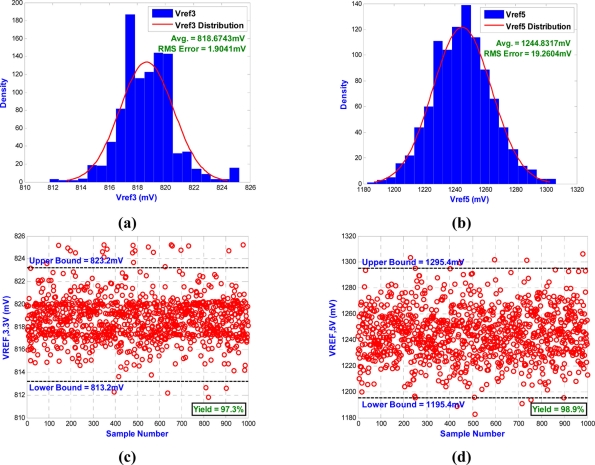
The results of the Monte Carlo analysis for **(a)** histogram of voltage references, *V*_*REF*,3.3*V*_ **(b)** histogram of voltage references, *V*_*REF*,5*V*_ **(c)** scatterplot of voltage references, *V*_*REF*,3.3*V*_ **(d)** scatterplot of voltage references, *V*_*REF*,5*V*_.

In Monte Carlo analysis, we assume that the W/L ratio of M1 and M2 in Figure 3 has a Gaussian distribution with 10% and three sigma process variations and 1,000 sample points. According to the simulation results, two voltage references can provide temperature-stable outputs of 818.67 mV and 1,244.83 mV with maximum deviations of 1.9 mV and 19.3 mV, respectively, as shown in Figure 6(a,b). Figure 6(c,d) shows the measured voltage reference points for each of the 1,000 sample points of the Monte Carlo run. The yield analysis of the voltage references for 3.3 V model indicates that 97.3% of the device samples will have a voltage reference within the required limit of the nominal value ±5 mV. The yield analysis of the voltage references for the 5 V model indicates that 98.9% of the device samples will have a voltage reference within the required limit of the nominal value ±50 mV.

### 3.2. Successive-Approximation-Register ADC (SAR ADC)

**Figure 7.**
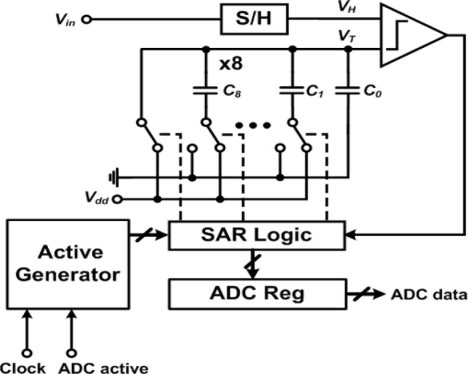
Block diagram of the successive approximation ADC.

Since the rate of temperature change is relatively slow, the SAR ADC is chosen for its lower area and the good ratio of speed/power [14]. Figure 7 shows the architecture of the SAR ADC that comprises a sample and hold (S/H), a comparator, a register array, a logic controller, and a binary-weighted capacitor array as a digital-to-analog converter (DAC) where C_8_ = 2C_7_, C_7_ = 2C_6_…, C_2_ = 2C_1_, and C_1_=C_0_. This ADC works as follows: first, the SAR sets the capacitor array to its middle scale (100…00) so that the output voltage *V_T_* of the DAC is *V_DD_*/2. Then the comparator compares the sample-and-hold (S/H) voltage *V_H_* with *V_T_* to determine whether the current setting is larger or smaller than *V_H_*. If *V_H_* > *V_T_*, the most significant bit (MSB) of SAR stays at logic 1, whereas if *V_H_* < *V_T_*, the MSB of SAR is set to logic 0. After the first bit is determined, the next bit is set to logic 1 (110…00 or 010…00), and the same procedure is repeated to determine the next bit. The sequence will be repeated eight times until all the bits are set.

**Figure 8.**
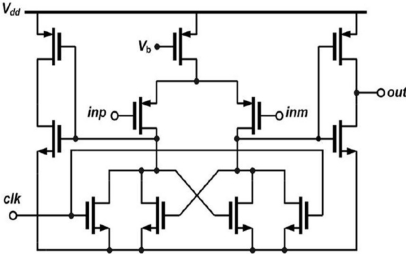
Schematic of the regenerative resettable comparator.

In order to lower the power consumption, the comparator used in this successive approximation ADC is a simple regenerative resettable comparator [15] followed by inverters for full-swing signal recovery, whose schematic is depicted in Figure 8. The comparator will be reset when the clock is high, while it will compare the DAC output voltage and input voltage when the clock is turned to low. Since the mismatch in the capacitance will seriously degrade the performance of the ADC, the capacitor array is arranged in the common-centroid configuration by using a relatively large unit capacitor of 400 fF. This ADC adopts a front-end passive S/H circuit composed of two switches and one capacitor (CH). The speed of the S/H circuit depends on the on-state channel-resistance (*R_on_*) of the switches, and the capacitance of CH. Since high-speed operation (or very small time constant) is unnecessary in this work, a relatively large CH of 21 pF (obtained by laying out as many capacitors as possible out of the available space) is adopted to reduce the conversion error. No power is dissipated in the S/H circuit due to its passive structure. The SAR is performed by the static CMOS logic circuit, which also generates the control signals such as the reset clock for the comparator and the sample clock for the S/H. The eventual digitized data converted by the ADC are stored in ADC registers.

### 3.3. On-Off Keying (OOK) Transmitter

**Figure 9.**
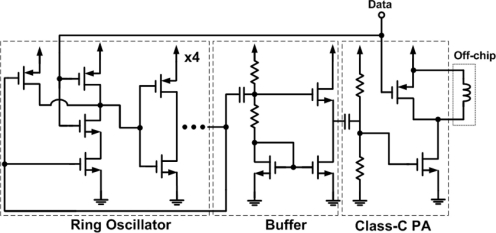
Circuit schematic of the transmitter.

In this work, the OOK modulation scheme was chosen for high throughput and high energy efficiency [16]. The circuit schematic of the OOK transmitter is depicted in Figure 9. It is composed of a ring-oscillator, a buffer (source follower), and a class-C power amplifier. The 433 MHz carrier signal is generated by the ring oscillator and the on-off keying modulation is achieved by turning the ring oscillator on and off. The Class-C power amplifier, consisting of an off-chip surface-mounted-device inductor, a large n-MOSFET, and a p-MOSFET switch, drives a sinusoidal current through the surface-mounted-device inductor, and in conjunction with the parasitic capacitance of the transistors/die pads to achieve a narrow band-pass frequency response in power amplifier. To prevent the data-rate degradation, a p-MOSFET switch is parallel-connected to the inductor, which accelerates the voltage decline at the output when the input signal changes from logic one to logic zero [17].

**Figure 10.**
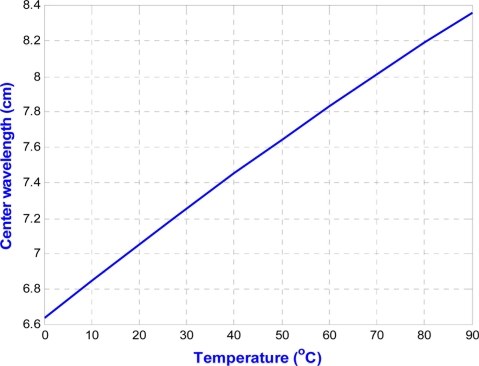
The relationship between the center carrier wavelength and temperature.

However, the oscillation frequency of this ring oscillator is very sensitive to temperature variation. As shown in Figure 10, the carrier center wavelength will change from 6.64 cm to 8.83 cm when the temperature varies from 0 °C to 120 °C. It is undoubtedly a big problem when applying this transmitter in wide-range temperature sensing application. Therefore, to overcome this problem, an off-chip phase locked loop (PLL) is replaced with the on-chip ring oscillator to provide a stable 433 MHz oscillation carrier in this phase. In the next version, a PLL circuit will be further integrated into this temperature chip.

### 3.4. Regulator

In our integrated wireless system, to prevent interference between each circuit block through the power line, several reference voltages and separate power supply sources are required. For example, we need a common mode voltage for the amplifier, a clean voltage source for the OOK transmitter and separate power supplies for the analog and digital circuits respectively, *etc.* Therefore, a circuit that can provide tunable voltage and enough driving ability is necessary in our design. Besides, a stable temperature-independent voltage supply circuit is essential because this temperature sensing system may operate in wide range of temperatures.

**Figure 11.**
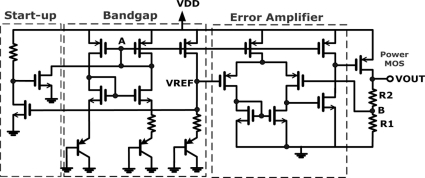
Schematic of the regulator.

The schematic of the regulator is shown in Figure 11 and consists of a start-up circuit, a bandgap reference, and an error amplifier. The function of the bandgap reference is to generate a temperature insensitive voltage (VREF) that is realized by typical current-mirroring bandgap reference topology. A start-up circuit is implemented to prevent the reluctance of the bandgap reference circuit from interfering the supply voltage. It helps to drag down the gate voltage at node A right after the supply voltage is applied, so that all transistors of bandgap reference can be biased correctly. The error amplifier is a simple single-ended op-amp architecture. Through negative feedback resistors (R1 and R2), the error amplifier will lock the voltage at node B to the reference voltage generated by the bandgap reference. Consequently, an output voltage (VOUT) of the regulator is also temperature-independent and given by:

(8) $$V_{\mathrm{OUT}}=V_{B}\cdot \frac{R1+R2}{R1}=\mathrm{VREF}\cdot \frac{R1+R2}{R1}$$

## 4. Experimental Results

### 4.1. Temperature Sensor

**Figure 12.**
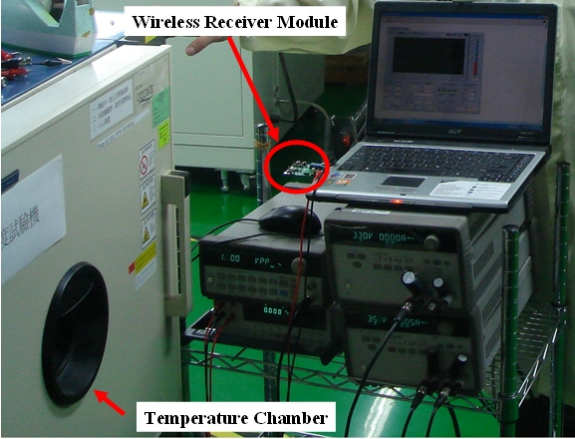
The experiment setup for temperature test on the packaged temperature sensor.

**Figure 13.**
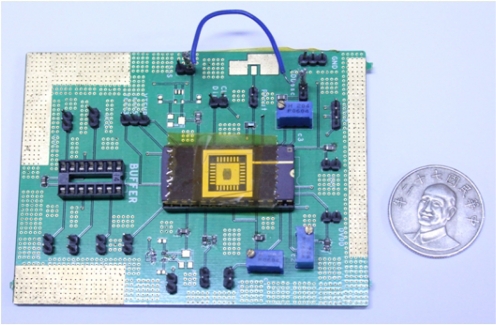
Photograph of the test PCB.

**Figure 14.**
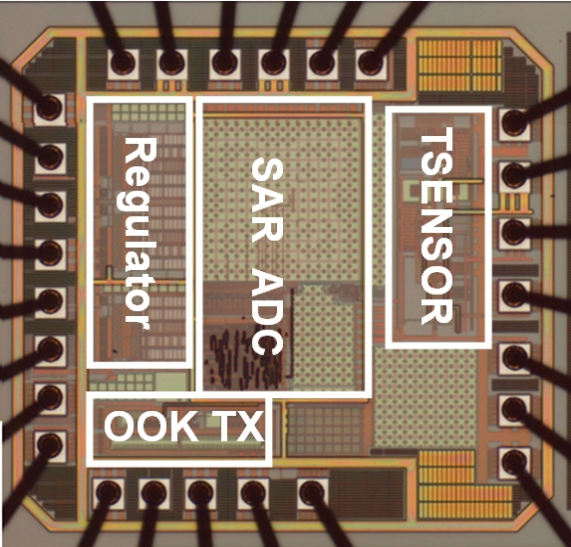
Micrograph for chip and wire bonding.

The proposed wireless temperature sensor has been tested in a MC-810 Mini-Subzero temperature chamber provided by Integrated Service Technology (IST) and the experimental setup has been established to test the sensitivity and linearity of the wireless temperature sensor, as shown in Figure 12. The red circle in Figure 12 highlights the wireless receiver module that acquires and stores immediate data from the wireless temperature sensor in Figure 1. A photograph of the test PCB block is given in Figure 13. The chip, shown in Figure 14, is 1.225 mm × 1.325 mm and contains the TSENSOR, SAR ADC, OOK TX, and Regulator blocks. Here, the TSENSOR and OOK TX mean the temperature sensor and OOK transmitter, respectively.

**Figure 15.**
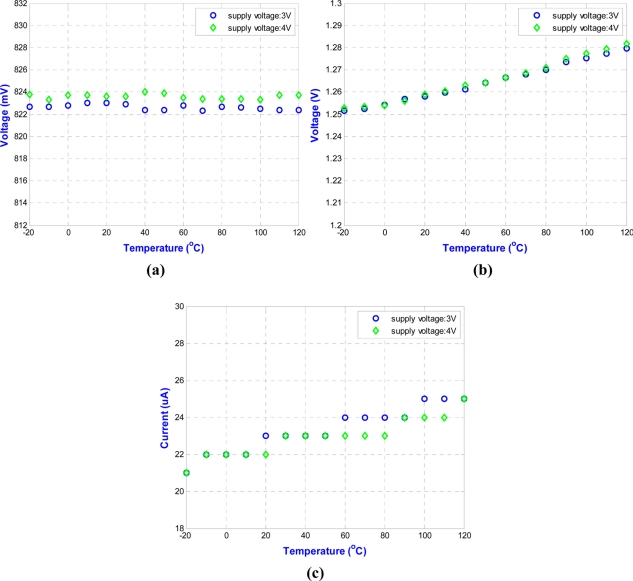
Measurement results for **(a)** voltage references, *V*_*REF*,3.3*V*_ **(b)** voltage references, *V*_*REF*,5*V*_, and **(c)** current reference, *I_REF_*.

**Figure 16.**
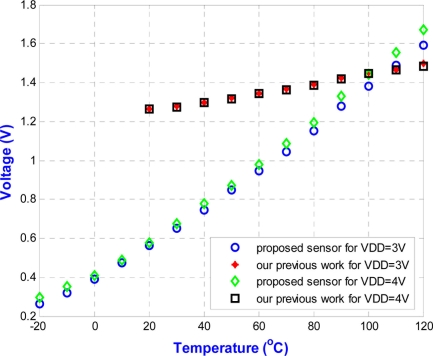
Comparison of the measured output voltage for the proposed temperature sensor and our previous work [8].

The circuit is fabricated using the 0.35 μm n-well double-poly CMOS process. The measurements use power supplies of 3.0 V and 4.0 V. The chip is measured in a temperature chamber by varying the temperature from −20 °C to 120 °C. For the voltage references, *V*_*REF*,3.3*V*_, *V*_*REF*,5*V*_, and the current reference, *I_REF_* are plotted in Figure 15. The two voltage references in Figure 15(a,b) can provide temperature-stable outputs of 823 mV and 1,265 mV with maximum deviations of 0.2 mV and 8.9 mV, respectively. The result for the current reference in Figure 15(c) gives a measurement of 23.5 μA, with a maximum deviation of 1.2 μA. The blue circle dots in Figure 16 show the variation of output voltage of the temperature sensor for power supply of 3.0 V. Thus, the sensitivity and linearity of the proposed wireless temperature sensor are 9.55 mV/°C, and 97%, respectively. The measurement results of the sensor in Figure 16 have 4.15-times better sensitivity than the previous design. The comparison of these two temperature sensors is shown in Figure 16. The variation of supply voltage has little effect on the design.

### 4.2. Wireless Transmitter

**Figure 17.**
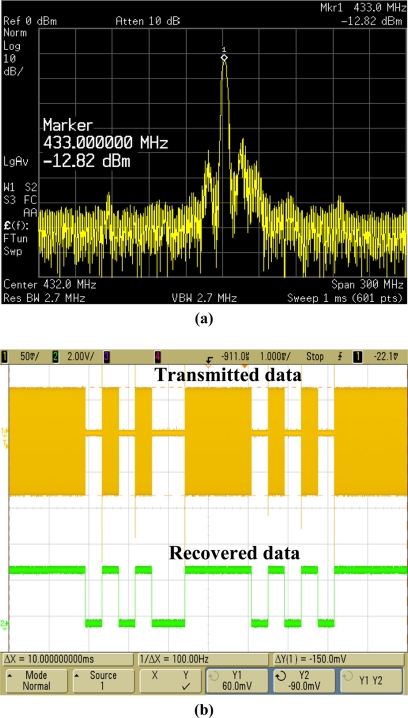
**(a)** Spectrum of the output power of the OOK transmitter. **(b)** Transmitted (by the transmitter) and recovered (by the receiver) temperature data observed by the oscilloscope.

Figure 17(a) shows the spectrum of the output power of the wireless OOK transmitter measured by using an Agilent E4440A PSA spectrum analyzer. The maximum power is about −13 dBm centered at 433 MHz, which is matched to the license-free ISM band. In our experimental setup, a self-designed low-power CMOS OOK receiver chip with sensitivity of −62 dBm and an antenna with 2 dBi gain are combined together to pick up the wireless transmitted data. During experiments, the distance between the transmitter and the receiver module is about 2 m and the data transmission looks quite stable. Furthermore, the maximum transmission distance in free space can be roughly calculated to be about 25 m according to the Friis transmission equation shown below:

(9) $$\frac{P_{r}}{P_{t}}=G_{t}\cdot G_{r}\cdot {\left(\frac{\lambda }{4\pi R}\right)}^{2}$$

where *P_r_* is available power at the output of the receiving antenna (receiver sensitivity), *P_t_* is power input to the transmitting antenna (output power of the transmitter), *G_t_* and *G_r_* are the antenna gains of the transmitting and receiving antennas respectively, λ is the wavelength, and *R* is the distance between the antennas. It is worthy to mention that the transmission distance could be further extended if we replace the self-designed receiver with commercial OOK receiver ICs, which usually have receiving sensitivity of −110 dBm.

**Figure 18.**
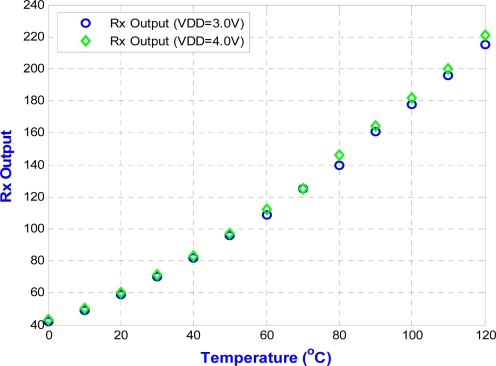
RX output with temperature signal processing.

The corresponding transmitted waveform with OOK modulation is shown in Figure 17(b). After being demodulated by the OOK receiver module, the original serial data can be successfully recovered and fed into the data collectors (laptops in this work). To further facilitate the recording process, a simple graphical user interface (GUI) based on LabVIEW software was developed. The blue circle dots in Figure 18 show the RX output for a power supply of 3.0 V. In addition, the linearity of the RX output of the proposed wireless temperature sensor is 98%, but the temperature range is only from 0 °C to 120 °C. At subzero temperatures, the output results of the RX output are not very linear because the on-chip OOK transmitter used here cannot be operated normally under such temperatures. From the chip results measured from −20 °C to 120 °C, the linearity of the proposed wireless temperature sensor is 97%. A performance summary is given in Table 1.

## 5. Conclusions

A combined device with two voltage references, one current reference, and a temperature sensor was created by using the DZTC principle and fabricated using the TSMC 2P4M process. From the chip results measured from −20 °C to 120 °C, the voltage references can provide a temperature-stable output at 823 mV and 1,265 mV. The temperature sensor has sensitivity and linearity up to 9.55 mV/°C, and 97%, respectively. For comparison, our previous PTAT temperature sensor only had sensitivity and linearity of 2.3 mV/°C, and 95%, respectively. In other words, the proposed sensor improves the sensitivity of our previous design 4.15-fold. The temperature sensor operates from −20 °C to 120 °C, achieving a resolution of 0.46875 °C, and ±0.6 °C inaccuracy without calibration. According to the experimental results, we can see that the proposed wireless temperature sensor has good precision and repeatability. The experimental results show that the immediate sensing temperature data can be successfully transmitted to the data collector through wireless communication. The combined device can also be fabricated using the CMOS process, which is suitable for embedded SoC applications.

## Figures and Tables

**Table 1.** Performance Summary.

Process	0.35 μm n-well double-poly CMOS
Chip size	1.62 mm^2^
***Temperature Sensor***	

Supply voltage	3 V–4 V
Temperature range	−20 °C to 120 °C
Sensitivity	9.55 mV/°C
Linearity	97%
Resolution	0.46875 °C
Inaccuracy	±0.6 °C
*V*_*REF*,3.3*V*_	823 mV
*V*_*REF*,5*V*_	1,265 mV
Current reference	23 μA
***SAR ADC***	

Resolution	8 bits
Power consumption	156.7 μW @ 2.4 kbps
***OOK Transmitter***	

Operating frequency	433 MHz
Output power	−12.8 dBm
Power consumption	11.9 mW @ 3 V
***Regulator***	

Line regulation	<80 mV/V
Load regulation	<66 μV/mA
Output to temperature variation	<4.67 μV/°C
